# Positive Effects of a Healthy Snack (Fruit) Versus an Unhealthy Snack (Chocolate/Crisps) on Subjective Reports of Mental and Physical Health: A Preliminary Intervention Study

**DOI:** 10.3389/fnut.2014.00010

**Published:** 2014-07-16

**Authors:** Andrew Paul Smith, Rosannagh Rogers

**Affiliations:** ^1^School of Psychology, Cardiff University, Cardiff, UK

**Keywords:** snacks, wellbeing, fruit, chocolate, crisps, depression, cognitive difficulties

## Abstract

**Background/Aims:** Recent research has shown associations between type of snack and wellbeing. These studies have been cross-sectional and the aim of the present research was to examine this topic using an intervention study.

**Methods:** A between-subjects intervention study was carried out. Volunteers (100 students, mean age = 19.00 years; 27 male, 73 female) completed online questionnaires measuring anxiety and depression, fatigue, somatic symptoms, cognitive difficulties, and distress at baseline. They were then randomly assigned to one of two snacking conditions – chocolate/crisps or fruit. Volunteers consumed one snack item in the mid-afternoon each day for 10 days. At the end of the intervention, the volunteers completed the questionnaires again.

**Results:** Analyses of the baseline data confirmed that consumption of chocolate was associated with greater emotional eating and depression. Analyses of covariance, with the baseline data as covariates, were carried out on the post-intervention responses. The results showed that consumption of fruit was associated with lower anxiety, depression, and emotional distress than consumption of crisps/chocolate. Similarly, scores for somatic symptoms, cognitive difficulties, and fatigue were greater in the crisps/chocolate condition.

**Conclusion:** These results extend findings from cross-sectional studies and give a clearer indication of causal effects of different types of snacks on wellbeing.

## Introduction

The study described in this article is part of our program of research on snacking and behavior. The research started by collecting data on the definition of snacking. One definition of snacking refers to eating a light meal or eating between meals. Our research has shown that eating several smaller meals at frequent intervals (“grazing”) is associated with better cognitive performance and mood than eating fewer and larger meals at longer intervals (1). A more usual definition of snacking refers to eating and drinking between meals (2). Our research has extensively investigated effects of beverages consumed between meals [see Ref. (3–5)]. Knowledge of the effects of snack foods is more restricted and our recent studies have aimed to rectify this.

Studies of the acute effects of snacks have shown similar effects to those observed after meals [e.g., effects of cereal bars are similar to those of breakfast – (6, 7)]. Smith (8) used methodologies developed in studies of meals on behavior to assess effects of regular patterns of snacking. These analyses showed little effect of snacking in general and this reflected the opposite effects of different kinds of snacks (9). The clearest example of this was found in a study of over 800 nurses (10). In this study, snacking on crisps, chocolate, and biscuits was associated with higher stress, more cognitive failures, and more injuries outside of work.

Many of the studies investigating effects of snacking have been cross-sectional. This means that it is unclear whether snacking changes mental health, or whether a person’s mental health influences their eating behavior. Research has repeatedly demonstrated that the experience of negative emotions such as sadness and stress, results in increased food consumption (emotional eating) and increased amount of unhealthy content (11, 12). A range of empirical evidence has provided strong support for the relationship between emotional state and eating. For example, controlled laboratory-based studies of stress induced eating (13, 14), self-report questionnaires (15), large population studies with children (16), and studies investigating the effects of naturally occurring stressful events such as examinations (17), have all provided strong support for the relationship between emotional eating traits and food consumption. Consumption of chocolate has often been related to emotional eating (18, 19). However, the debate about causality often applies to chocolate consumption. Correlations have been found between higher levels of depression in those who rely on chocolate to increase their moods (20), people who crave chocolate more (21), and those who label themselves as “chocolate addicts” (22). However, it is unclear whether those who are depressed eat more chocolate (emotional eating), or whether chocolate consumption is playing a role in maintaining or causing mental health problems. Both views have some support. For example, Parker and colleagues acknowledged that there are positive feelings experienced after eating chocolate in response to emotional eating; however, they also state that it is more likely that chocolate actually prolongs feelings of dysphoria (19). Emotional eating is not restricted to chocolate. For example, in a diary study Steptoe et al. (23) found more alcohol and fast food was consumed in high stress weeks.

There is also considerable support for the other causal pathway, namely that diet can influence mental health. Much of this research also involves cross-sectional studies. For example, a large number of correlational studies show that regular consumption of breakfast is associated with higher wellbeing scores [see Ref. (4, 24) for reviews]. These early results have been followed by intervention studies [e.g., Ref. (25, 26)], which have confirmed that consumption of breakfast is associated with greater wellbeing.

Research has also investigated other aspects of healthy eating, for example, consumption of fruit and vegetables. Results from large cross-sectional surveys show an association between positive aspects of wellbeing (life satisfaction and happiness) and greater consumption of fruit and vegetables (27). A recent daily diary study (28) has shown greater positive effect on days when more affect servings of fruit and vegetables were consumed. Results of lagged analysis showed that consumption of fruit/vegetables predicted increased wellbeing the next day, which suggests that it was consumption of healthy foods that was driving reported health not the other way around. Interventions have also examined effects of specific fruit (e.g., gold kiwifruit, a high vitamin C food) on mood (29).

The purpose of the present study was to conduct an intervention study comparing consumption of unhealthy (chocolate/crisps) and healthy (fruit) snacks on subjective reports of mental and physical health. It followed the approach adopted in our breakfast research and used a 10-day intervention period. This duration is short enough to maintain compliance and has also been shown to be sensitive to dietary interventions [e.g., effects of fiber – (25)]. At baseline, correlations were computed to try to replicate previous results on associations between consumption of different snack types and subjective reports of health. The Dutch Eating Behavior Questionnaire (DEBQ) (30) was also administered at baseline to examine associations between emotional eating and consumption of different types of snacks. Following baseline, the snacking intervention was carried out for 10 days and further measures of subjective health taken at the end of this period.

## Materials and Methods

The study received approval from the School of Psychology ethics committee and was carried out with the informed consent of the participants.

### Design

A between-subjects design involving baseline measurements followed by a 10-day intervention was used. Cross-sectional data allowed investigation of associations between frequency of consuming different snacks, eating behavior and reports of physical and mental health. The intervention examined causal relationships between the health outcomes and snacking behavior. At enrollment, participants were given a number from 1 to 100. A random number generator selected 50 numbers from 1 to 100 and these participants were assigned to one condition and those participant numbers not selected assigned to the other. The dependent variables measured were anxiety, depression, emotional distress, somatic symptoms, fatigue, and cognitive difficulty (see below).

### Participants

#### Sample size calculation

Previous studies using this methodology have demonstrated effects of about 0.6 SD. With power set to 0.8, *p* set to 0.05, a sample size of 44 per group was required.

One hundred undergraduate psychology students (mean age = 19 years SD = 0.79: male = 27, female = 73; 98% white) completed this study. Participants were recruited from the School of Psychology Human Participant Panel and received course credits in exchange for participation.

#### Materials

Subjective reports of snack consumption, eating behavior, and health were measured using questionnaires presented on Survey Tracker. Survey Tracker exported all data straight into SPSS. Habitual consumption of chocolate (bars per week), crisps (bags per week), and fruit (pieces per week) were assessed at baseline. Subjective health was measured using the profile of fatigue related symptoms [PFRS; measures emotional distress, fatigue, somatic symptoms, and cognitive difficulties – (31)] and the hospital anxiety and depression scale [HADS; measures anxiety and depression – (32)]. Eating behavior was measured using the DEBQ (30). The main interest in the DEBQ was the emotional eating scale and only these data are reported here.

#### Snacks

In the fruit condition, participants were given 10 pieces of fruit (4 golden delicious apples, 3 large clementines, and 3 bananas) and told to consume 1 piece each afternoon for 10 days. In the chocolate/crisps condition, volunteers were given 10 snacks (5 packets of assorted crisps and 5 chunky chocolate wafers). Snacks were chosen on the basis that chocolate and crisps represent unhealthy snacking whereas fruit is associated with health snacking (9, 10, 18).

#### Procedure

Participants then filled out the first online questionnaire (baseline measures), which took 8 min and then were given their snacks. Written instructions informed participants to consume one provided snack once a day for 10 days, preferably in the afternoon as well as consume their normal diet. If participants were unable to store all of their snacks they took some and collected the rest during the 10-day period.

Participants were told that they would get a reminder email on the fifth day of the intervention, which gave details of when they would receive the second questionnaire and asked to email the experimenter if they were experiencing any problems with the study. On the ninth day, participants were sent the second questionnaire (secondary measures) and were told to complete it on the last day of the intervention (day 10) after they had consumed their last snack. When the baseline data and post-intervention data were merged the database was made anonymous, participants were credited for their participation, and debriefs were emailed to participants.

### Statistical analysis

Data analysis involved Pearson product moment correlations being computed for baseline measures of snacking frequency, wellbeing (PFRS; HADS) and eating behavior (DEBQ). This was to replicate previous results from cross-sectional studies. Analyses of covariance, with snacking condition as the between subject factor, were carried out on the post-intervention scores with the corresponding baseline score and emotional eating as covariates. Entering these factors into the statistical model allowed the analysis to assess if the intervention had a significant impact on wellbeing when controlling for emotional eating and baseline measures.

## Results

The two intervention groups did not differ on age, gender nor the baseline PFRS, HADS, or DEBQ variables (all *p*’s > 0.05).

### Correlations between baseline measures

Results revealed that emotional eating and depression correlated significantly with consumption of chocolate (Table 1). None of the other correlations were significant.

**Table 1:** **Correlations between regular consumption of healthy/unhealthy snacks, subjective reports of health and emotional eating in the baseline data**.

	Chocolate	Crisps	Fruit
Emotional distress	0.14	−0.06	−0.08
Fatigue	0.13	−0.06	−0.10
Cognitive difficulties	0.08	0.05	−0.14
Somatic	0.02	0.00	−0.08
Anxiety	−0.01	−0.08	0.05
Depression	0.19*	−0.09	0.00
Emotional eating	0.21*	−0.08	0.05

*p < 0.05, 1-tail.

### Results for the intervention

Analyses of covariance revealed that there were significant differences in subjective reports of health between the snacking conditions (see Table 2). The adjusted means demonstrate the effects of the intervention with those in the fruit condition reporting lower mental and physical health problems compared with those in the chocolate and crisps condition.

**Table 2:** **Adjusted mean scores (SES) for wellbeing scores of the two snack conditions (high scores = lower wellbeing)**.

Measure	Fruit	Chocolate/crisps	Significance
Anxiety	5.46 (0.33)	6.77 (0.33)	*F*(1,96) = 7.95, *p* < 0.05
Depression	2.40 (0.25)	3.32 (0.25)	*F*(1,96) = 6.43, *p* < 0.05
Somatic	28.10 (1.36)	33.82 (1.36)	*F*(1,96) = 8.26, *p* < 0.005
Cognitive	25.59 (1.21)	31.61 (1.21)	*F*(1,96) = 11.44, *p* < 0.001
Fatigue	27.83 (1.36)	34.21 (1.36)	*F*(1,96) = 10.33, *p* < 0.005
Emotional distress	36.89 (1.70)	46.57 (1.70)	*F*(1,96) = 16.11, *p* < 0.001

If one examines percentage change from baseline (Table 3) one finds that those in the chocolate/crisp condition reported large increases in depression, emotional distress, and fatigue. Other outcomes were lower than baseline in the chocolate/crisps condition but the reductions were significantly greater in the fruit condition.

**Table 3:** **Percentage change from baseline in wellbeing scores for the different snacking condition**.

	Fruit (%)	Chocolate/crisps (%)
Anxiety	−31.8	−18.5
Depression	−0.5	+46.6
Somatic	−21.7	−7.0
Cognitive	−15.0	−0.3
Fatigue	−8.7	+10.6
Emotional distress	−7.2	+10.2

## Discussion

Recent reviews [e.g., Ref. (33, 34)] and meta-analyses (35) highlight the need for further research on snacking and health. Despite many years of interest in the effects of snacking, there has been a lack of consistency in study design and definition of snacking. In addition, many of the studies have been cross-sectional and failed to provide control for potential confounders. There is evidence that snacking consisting of better diets (e.g., greater consumption of fruit and vegetables) is associated with better mental health (35–38). Again, most of these studies have been cross-sectional, which makes it difficult to assign causality.

The results from the present intervention study support the view that consumption of fruit improves mental health compared to consumption of crisps/chocolate. Further research is required to determine the extent to which these effects generalize across other types of snack. Indeed, even within the category of fruit there may be variation depending on the micronutrient composition of the product. Similarly, it has been suggested that dark chocolate may have potential benefits due to anti-oxidant and phytochemical effects (39). However, results from a double blind placebo controlled study failed to demonstrate beneficial effects of dark chocolate (40).

The present study was intended to be an initial intervention and there were some limitations in the methodology. First, it would have been desirable to collect a more detailed dietary intake both at baseline and at the end of the intervention. Although participants were instructed to maintain their normal diet, it is possible that the effects reported here may reflect factors other than the intervention. It would also be useful to examine daily reports of mental and physical health in order to determine how rapidly effects of the intervention develop. Finally, it is important to include measures of positive affect and other aspects of wellbeing in future research.

The current study tells us little about the mechanisms underlying the effects of the different snacks on wellbeing. Two types of explanation can be distinguished. The first type of effect might focus on the nutrients provided by the different snacks. In contrast to this, it may be the cognitions associated with the different snacks that are important. This last view could be tested by giving volunteers identical snacks but labeling one as “unhealthy” and the other as “healthy.”

In conclusion, the results from the present intervention study confirm and extend earlier cross-sectional research on the effects of different types of snack on subjective reports of health [e.g., Ref. (9)]. Mental health is important in its own right but it is also a major risk factor for chronic disease (41). Given that many mental health problems are initially apparent in adolescence and early adulthood (42), it is now important to examine whether common mental disorders can be prevented by simple dietary interventions.

## Conflict of Interest Statement

The authors declare that the research was conducted in the absence of any commercial or financial relationships that could be construed as a potential conflict of interest.
